# Implementing a continuous quality-improvement framework for tuberculosis infection prevention and control in healthcare facilities in China, 2017–2019

**DOI:** 10.1017/ice.2023.287

**Published:** 2024-05

**Authors:** Canyou Zhang, Stephanie O’Connor, Sarah E. Smith-Jeffcoat, Diana Forno Rodriguez, Hui Guo, Ling Hao, Hui Chen, Yanbo Sun, Yan Li, Jiying Xu, Liang Chen, Lan Xia, Xing Yang, Anand Date, Jun Cheng

**Affiliations:** 1 National Center for Tuberculosis Control and Prevention, Chinese Center for Disease Control and Prevention, Beijing, China; 2 Global Health Center, US Centers for Disease Control and Prevention, Atlanta, Georgia, United States; 3 CSL Behring Beijing Office, Beijing, China; 4 US Centers for Disease Control and Prevention China Office, Beijing, China; 5 Heilongjiang Provincial Center for Disease Control and Prevention, Harbin, Heilongjiang, China; 6 Jiangsu Provincial Center for Disease Control and Prevention, Nanjing, Jiangsu, China; 7 Henan Provincial Center for Disease Control and Prevention, Zhengzhou, Henan, China; 8 Guangdong Provincial Institute of Public Health, Guangzhou, Guangdong, China; 9 Sichuan Provincial Center for Disease Control and Prevention, Chengdu, Sichuan, China; 10 Yunnan Provincial Center for Disease Control and Prevention, Kunming, Yunnan, China

## Abstract

**Background:**

Tuberculosis (TB) infection prevention and control (IPC) in healthcare facilities is key to reducing transmission risk. A framework for systematically improving TB IPC through training and mentorship was implemented in 9 healthcare facilities in China from 2017 to 2019.

**Methods:**

Facilities conducted standardized TB IPC assessments at baseline and quarterly thereafter for 18 months. Facility-based performance was assessed using quantifiable indicators for IPC core components and administrative, environmental, and respiratory protection controls, and as a composite of all control types We calculated the percentage changes in scores over time and differences by IPC control type and facility characteristics.

**Results:**

Scores for IPC core components increased by 72% during follow-up when averaged across facilities. The percentage changes for administrative, environmental, and respiratory protection controls were 39%, 46%, and 30%, respectively. Composite scores were 45% higher after the intervention. Overall, scores increased most during the first 6 months. There was no association between IPC implementation and provincial economic development or volume of TB services.

**Conclusions:**

TB IPC policies and practices showed most improvement early during implementation and did not differ consistently by facility characteristics. The training component of the project helped increase the capacity of healthcare professionals to manage TB transmission risks. Lessons learned here will inform national TB IPC guidance.

Tuberculosis (TB) infection prevention and control (TB IPC) is a primary tool in reducing TB transmission, particularly in known hotspots like healthcare facilities.^1^ TB IPC is particularly important where TB burden is high. China has the third highest TB burden, with an estimated 748,000 new TB patients in 2022.^2^ Also, 20% of all reported TB cases among healthcare workers (HCWs) globally in 2022 were from China.^3^ Multiple subnational studies in China have found the prevalence of latent TB infection (LTBI) in HCWs to be >50%,^4–7^ which may indicate ongoing nosocomial transmission. Other studies have found lower but substantial levels of LTBI among HCWs in China, ranging from 22% to 34%.^8–10^ In a cross-sectional study of 31 health facilities in China, regular facility-based IPC training and implementation was associated with low HCW LTBI prevalence,^10^ highlighting the importance of facility IPC policies.

The TB Building and Strengthening Infection Control Strategies (TB BASICS) is a framework designed to prepare public health professionals (1) to rapidly and thoroughly assess a facility’s TB IPC policies and practices, (2) to develop and implement a plan for improving TB IPC at the facility level, and (3) to then monitor for continuous quality improvement. IPC training and ongoing mentorship are key elements throughout the framework to promote capacity building and sustainability. The backbone of TB BASICS is the hierarchy of controls, which categorizes IPC measures as administrative, environmental, or respiratory protection controls.^11,12^ TB BASICS has been conducted in 18 countries around the world, including Zambia and Botswana, where facility assessments showed an increase in IPC performance scores and notable improvements in triage and fast-tracking of coughing patients, active TB screening, and provision of N95 respirators.^13,14^

The Chinese Center for Disease Control and Prevention (China CDC), with technical assistance from the US Centers for Disease Control and Prevention (US CDC), conducted an 18-month pilot program of TB BASICS in 9 TB referral hospitals in China from 2017 to 2019. The goal of the program is to promote lasting change in TB IPC practices in healthcare settings. We describe the TB BASICS framework as it was implemented in China and report trends in IPC implementation scores during the project period. The analysis examines differences in IPC scores by IPC control type and by facility characteristics such as provincial economic development level and volume of TB services delivered.

## Methods

### Project description

TB BASICS was implemented in 9 healthcare facilities in China from September 2017 to July 2019. The National Center for TB Control and Prevention (NCTB) purposively selected facilities of varying sizes in 2 high-development provinces, 2 middle-development provinces, and 2 low-development provinces (Table 1). Development level reflected an existing classification from the National Bureau of Statistics that is based on economic factors.^15^ Results were analyzed by provincial development level to better understand possible correlation between IPC implementation and surrounding economic conditions. Facilities were classified as having a small, medium, or large TB service delivery volume based on the number of TB outpatient visits in the year before TB BASICS implementation (range, 94–78,521). Facilities with <2,000 TB outpatient visits were considered small. Medium-sized facilities had 2,000–20,000 outpatient visits, and facilities reporting >20,000 visits were considered large.

**Table 1.** Characteristics of Participating Facilities

Provincial Development Level^a^	Facility Number	TB Services Volume^b^	Inpatient Departments	Outpatient Departments	Laboratory Departments	No. (%)
High	1	Large	3	2	1	6 (11)
	2	Medium	0	4	1	5 (9)
	3	Medium	1	4	1	6 (11)
	Subtotal by development level					17 (30)
Middle	4	Large	8	3	1	12 (21)
	5	Medium	0	2	2	4 (7)
	6	Large	9	3	1	13 (23)
	7	Small	0	1	1	2 (4)
	Subtotal by development level					31 (54)
Low	8	Small	1	4	1	6 (11)
	9	Small	1	1	1	3 (5)
	Subtotal by development level					9 (16)
Subtotal by Department Type (%)			23 (40)	24 (42)	10 (18)	57

a Provincial development level was determined by the National Bureau of Statistics.

b No. of TB outpatient visits was used as a proxy for TB service volume. Small, <2,000 TB outpatient visits; medium, 2,000–20,000 TB visits; large, >20,000 TB visits.

Each facility and provincial CDC identified staff to form a facility-level core team comprising 2 provincial CDC representatives and 8 staff members from each facility, including 2 from the infectious disease unit, 2 from the outpatient unit, 2 from the inpatient unit, 1 laboratory technician, and 1 radiologist. TB IPC specialists from the NCTB and US CDC conducted TB IPC training for core team members (12 provincial and 72 facility-level), who completed knowledge evaluations before and after the training.

Following the initial IPC training, NCTB specialists led baseline assessments at every facility using standardized tools. IPC core components were assessed at the facility level, and administrative, environmental, and respiratory protection controls were evaluated within individual departments. The number of departments assessed varied among facilities and ranged from 2 to 13 (Table 1). All departments involved in TB screening, diagnosis, and treatment were included in the project, but some departments joined after the baseline assessment.

**Figure 1.**
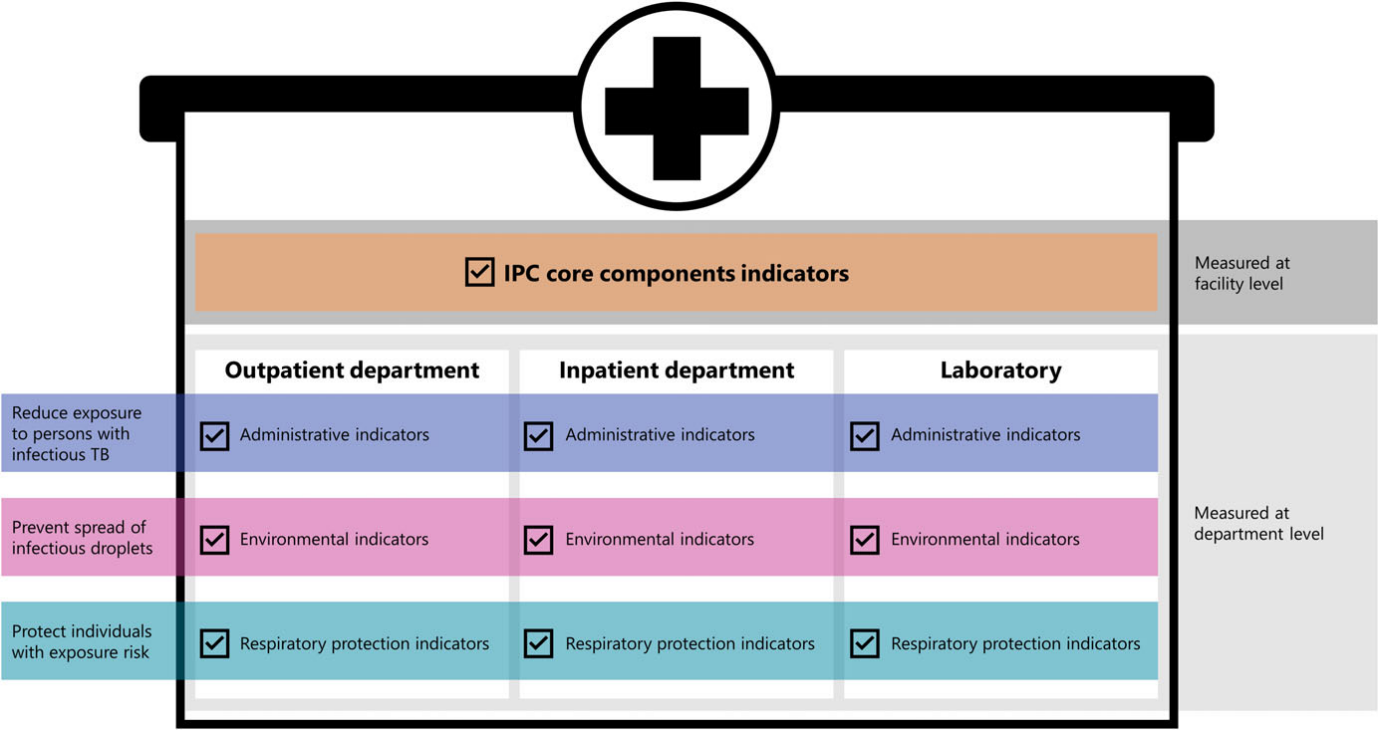
Infection prevention and control assessment structure.

The facility-level assessment tool included 16 IPC core-component indicators evaluated based on document review and interviews. This tool included indicators such as the presence of an active IPC committee and policies supporting TB screening for staff members. The department-specific tools for outpatient, inpatient, and laboratory had 34, 33, and 20 indicators, respectively (Supplementary Material 1 online). The department-specific assessment tools were subdivided into administrative, environmental, and respiratory protection controls (Fig. 1). Laboratory assessments were for TB IPC only and did not include a comprehensive biosafety evaluation. Some indicators assessed IPC measures that require little or infrequent maintenance once in place, such as conducting annual fit testing. Most indicators were phrased to determine whether the measure was sustained throughout follow-up, and ongoing monitoring of all indicators was an important component of the project.

The assessment tools were developed based on previous TB BASICS projects and World Health Organization guidance^16^ and were further adapted to the local setting. Thorough standard operating procedures for the assessment tools aimed to promote standardization by using objective measures to evaluate IPC indicators when possible. Quantifiable indicators were considered fully implemented if they were observed in >80% of instances. Partial implementation was 40%–80%, and any indicator below 40% was considered not implemented.

Based on baseline assessments, core teams developed evidence-based and measurable facility IPC intervention plans targeting identified weaknesses. Plans were implemented with local and national support and included funding, supplies, regular onsite and remote mentoring, and technical assistance from NCTB TB IPC specialists. Follow-up assessments were conducted quarterly by the local China CDC and core teams for 18 months. Facilities were provided with color-coded dashboards (Supplementary Material 2 online) to help track progress. Local supervisory teams and the focal person from the NCTB provided monthly mentoring for core teams. Facility staff who were not part of a core team were trained to identify and address challenges as they arose to promote a culture of self-monitoring. The final reassessment was conducted jointly by each facility’s core team and specialists from the NCTB and the US CDC.

### Data collection

At baseline and every quarterly follow-up, core teams evaluated TB IPC policies and practices using up to 4 different assessment tools. The facility-level assessment tool was completed at all facilities and assessed managerial or administrative TB IPC controls, now referred to as IPC core components.^1^ Each of the other 3 assessment tools was tailored to evaluate a different department involved in TB diagnosis and treatment: inpatient, outpatient, or laboratory. For these 3 tools, 1 assessment was completed per participating department. Sites shared results with NCTB to seek feedback on TB IPC. Data were stored in Excel spreadsheets (Microsoft, Redmond WA).

### Data analysis

Facility-based TB IPC implementation assessments were conducted quarterly, and scores were calculated and shared via dashboards: 2 points were assigned for each fully implemented indicator, 1 point if partially implemented, and no points if not implemented. Scores were calculated as a percentage by dividing points earned by the maximum possible score. Scores ≥80% were generally considered acceptable IPC implementation. Not all indicators were universally applicable, so the number of indicators differed by facility and department. Percentage change was calculated by dividing the difference between scores at 2 time points by the score from the earlier time point.

For each of the 4 control types (ie, core components, administrative, environmental, and respiratory protection), scores of all participating departments within a facility were averaged to produce scores by control type. These 4 scores were then averaged to produce a composite score representing the whole facility. For analysis by development level, composite scores were then averaged, resulting in 1 score for all facilities in provinces within each development level. The same method was used to calculate average scores by volume of TB services. The association between composite score and time from baseline as well as the effect of development level and volume of TB service delivery on composite score were examined using mixed-effects linear regression models adjusted for time from baseline and with a random intercept for each facility to account for facility-level variability. We used a Wald χ^2^ test to generate *P* values; statistical significance was set at α = 0.05.

## Results

Participating facilities completed 62 IPC core components evaluations over 18 months. Each facility completed an average of 6 department-level assessments per quarter (range, 2–12). Because additional departments were added after the baseline assessment, the number of participating departments fluctuated from 45 to 57 during follow-up (Table 1). One facility underwent infrastructure changes that prohibited its participation in the postintervention assessment.

### IPC core components scores

**Figure 2.**
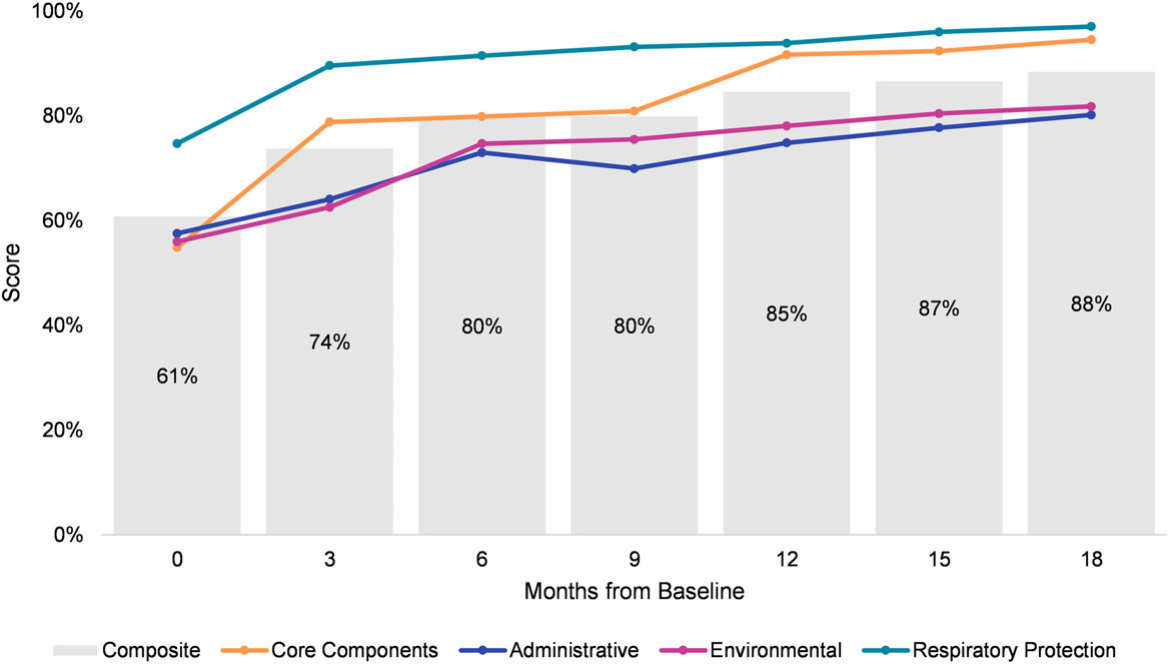
Infection prevention and control assessment scores by control type.

**Figure 3.**
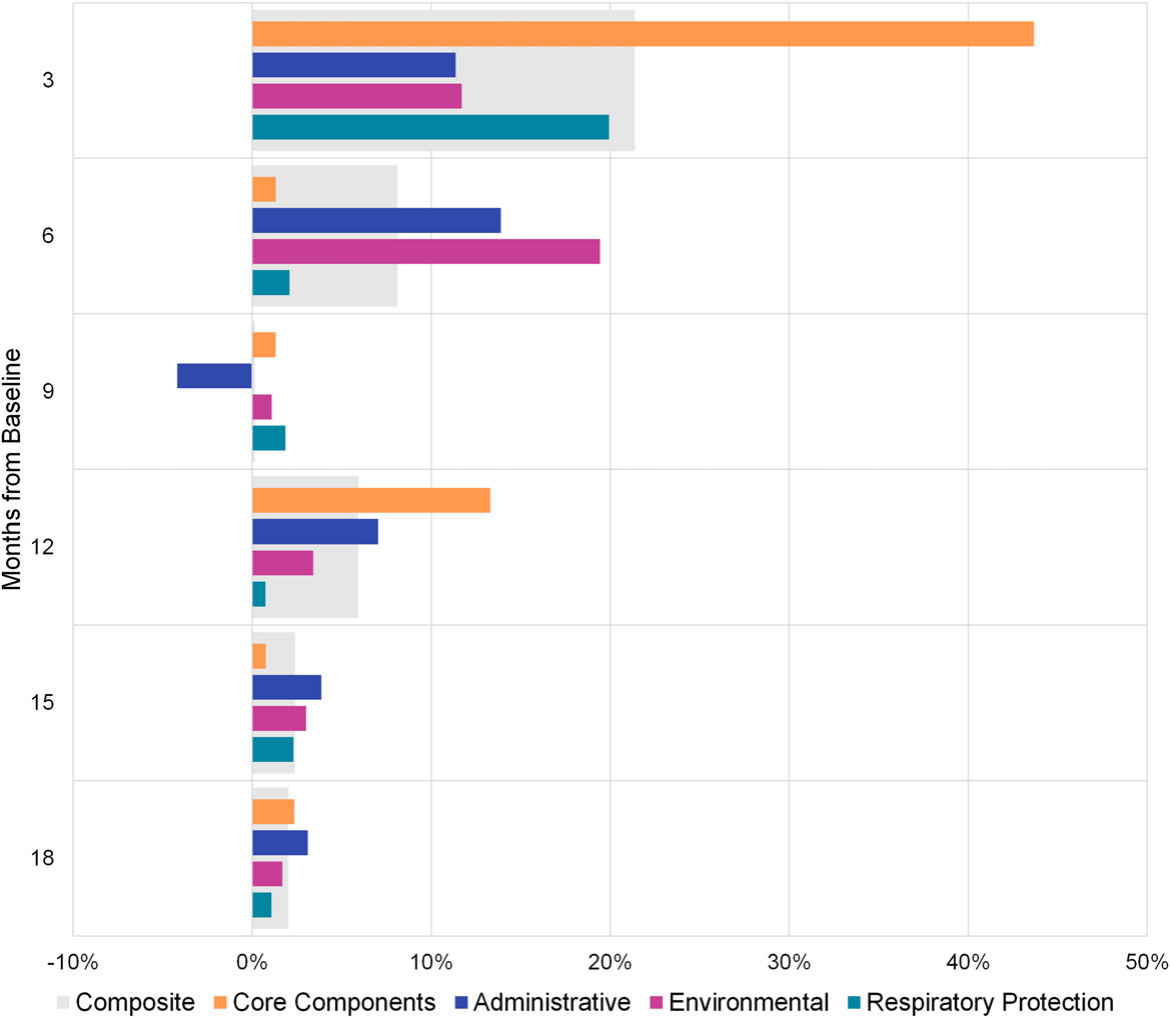
Percent change in infection prevention and control assessment scores from previous quarter by control type.

**Figure 4.**
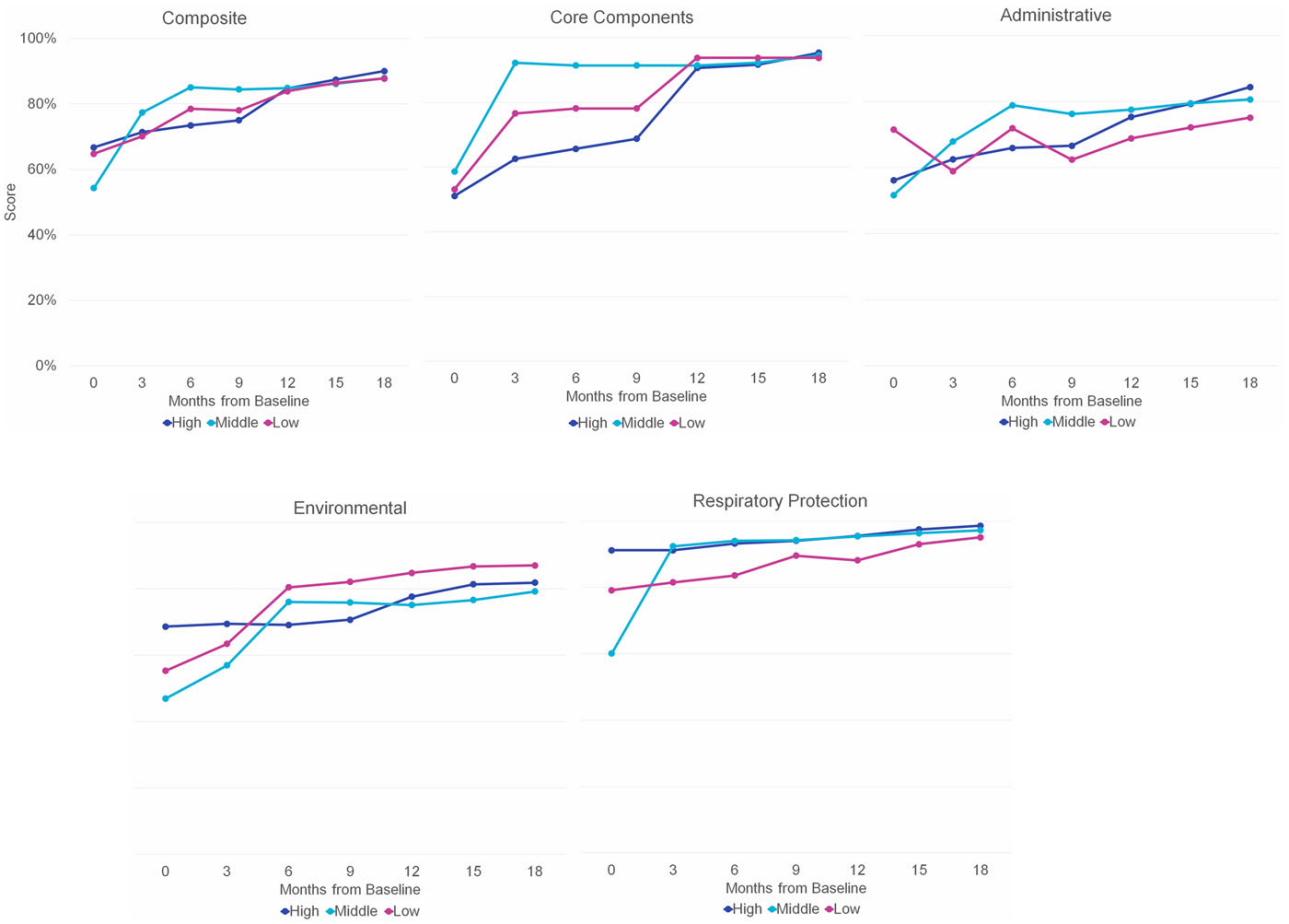
Infection prevention and control assessment scores by control type and development level.

The mean score for the 16 core-component indicators was 55% at baseline (Fig. 2), suggesting that most recommended IPC policies and practices were partially implemented or were not in place. At the first follow-up, the mean increased by 44% (Fig. 3). Overall, the mean score was 72% higher at the final evaluation than at baseline. At baseline, IPC core-component scores were similar by development level but were highest among facilities in middle-development provinces (Fig. 4). At the final evaluation, scores were nearly identical across development levels, with facilities in high- and middle-development provinces scoring 95% and those in low-development provinces scoring 94%.

### Administrative, environmental, and respiratory protection scores

Respiratory protection controls were consistently implemented at higher rates than other controls. Mean scores at baseline were 58%, 56%, and 75% for administrative, environmental, and respiratory protection controls, respectively (Fig. 2). For administrative and environmental controls, there was a substantial positive percent change in scores in the first 6 months (Fig. 3). After the intervention, the mean scores were 80% for administrative, 82% for environmental, and 97% for respiratory protection controls, indicating strong implementation of gold-standard infection prevention measures.

Scores for administrative IPC measures were highest among facilities in low-development provinces at baseline (Fig. 4). By the final assessment, facilities in high-development provinces had the highest average administrative score at 84%, followed by middle-development provinces at 81% and low-development provinces at 75%.

For environmental controls, facilities in high-development provinces had the highest baseline average of 69% (Fig. 4). Both low- and middle-development provinces had substantial gains in the first 2 quarters, with increases of 46% and 62% from baseline, respectively. At the final evaluation, the highest mean score was among facilities in low-development provinces at 87%, compared with 82% for facilities in high-development provinces and 79% for middle-development provinces.

The widest baseline gap between development levels was noted for respiratory protection indicators (Fig. 4). Facilities in high-development provinces had a mean score of 91%, followed by low-development provinces at 79% and middle-development provinces at 60%. By the postintervention assessment, facilities in high-development provinces still scored the highest at 99%, followed by middle-development provinces at 97% and low-development provinces at 95%.

### Composite scores

The mean composite score, comprising IPC core components, administrative, environmental, and respiratory protection scores, was 61% at baseline (Fig. 2). The largest improvements occurred during the first quarter, when there was a 21% positive change in the mean (Fig. 3). At the final evaluation, the mean composite score was 88%, representing a 45% increase from baseline. The difference between baseline and postintervention composite scores was statistically significant (Wald test, *P* < .001), with scores increasing by an average of 25% after 18 months. Mean composite scores >80% were reported from the 6-month mark onward, suggesting that facilities had adopted and maintained robust infection prevention practices.

Facilities in high-development provinces had the highest mean baseline composite score at 67%, followed by facilities in low-development provinces at 65% and middle-development provinces at 54% (Fig. 4). By the final evaluation, scores were similar across development levels at 90% for high-development provinces and 88% for low- and middle-development provinces. No association was detected between composite score and provincial development level.

When using TB outpatient visits as a proxy for volume of TB services, there was no statistically significant association with composite score. Baseline scores by TB services volume ranged from 58% to 65%. At the postintervention evaluation, composite scores ranged from 83% to 95%, with large-volume facilities ranking lowest and medium-volume facilities ranking highest.

## Discussion

IPC implementation improved throughout the follow-up, but progress was generally concentrated in the first 2 quarters. This finding suggests that 6 months was sufficient to make notable improvements in IPC practices and policies. Slower progress after 6 months reflects achievement of exceptionally high IPC standards at some facilities and highlights the resource-intensive nature of some IPC measures that were not fully implemented even after 18 months of follow-up, such as having openings on opposing walls for natural ventilation. Part of the early increase in scores may have been due to the adoption of controls that could be considered self-sustaining or required minimal ongoing effort, such as having a facility-specific IPC plan. Sustained score improvements over an 18-month period suggest that these measures remained in place long after the initial IPC training.

Because IPC core components are managerial measures, they may be more sensitive to attention from facility leadership than funding inputs, which may explain why even facilities in low-development provinces showed great improvement in this area. Environmental controls requiring infrastructure changes may explain score stagnation following initial improvements. Many environmental controls require substantial investments that could take years to design, fundraise, and implement; therefore, these changes may not have occurred within the project timeline.

Although facilities in high-development regions did average higher scores in some analyses, there was not a clear correlation between development level and TB IPC implementation. And although there was a wider score distribution for administrative and environmental controls, the rank order of development level scores was not consistent. These findings suggest that development level is not an insurmountable barrier to proper IPC implementation, highlighting the role of other factors in addition to resource availability, such as institutional attitudes about IPC.

### Successes

Many factors contributed to successful implementation of TB BASICS in China. Representatives from NCTB and US CDC worked to gain the support of facility and department leadership to ensure stakeholder buy-in. Supplemental funding and materials, such as respiratory protection supplies, were available to participating facilities when needed. Existing TB BASICS tools used in other settings were adapted to make indicators measurable and as objective as possible. Ongoing mentorship was a key component of the project, with in-person technical and supervisory support and in-person training. Experience-sharing sessions were held throughout implementation, and the NCTB hosted a chat group for brainstorming solutions in real time. Online resources were made available for training, assessment, and continuous quality improvement. For facilities in underdeveloped areas, project implementors focused on solutions to maximize limited resources and consistent implementation of funding-independent IPC measures.

The impact of the TB BASICS pilot program in China reached beyond the 9 participating facilities. The assessment tool package has been incorporated into the monitoring and evaluation section of China’s updated national TB IPCC guidelines published in November 2023.^17^ By reinforcing IPC measures that help prevent respiratory disease transmission, drawing awareness to the importance of IPC, and developing transferrable IPC skills, the project inadvertently prepared healthcare facilities to better manage COVID-19. Chongqing Public Health Medical Center, a pilot participant in TB BASICS, was designated for treatment of COVID-19 patients in January 2020. The facility’s management of COVID-19 transmission risks was strongly informed by TB BASICS principles and practices.^18^

This study had several limitations. The pilot program included only TB-designated hospitals, and it may be more difficult to implement TB BASICS in general hospitals, where separation of TB patients may be particularly challenging. However, TB BASICS may be helpful in preparing for any number of other infectious diseases like COVID-19 and influenza. Some departments were evaluated for the first time after the baseline assessment. For example, at the 9-month follow-up, 1 facility assessed 3 additional departments, all of which received low administrative control scores, which contributed to the decrease observed at that time point (Fig. 3). However, in a sensitivity analysis in which all departments that did not participate in the baseline assessment were excluded, composite scores were <2% different at each quarterly assessment, suggesting that including a relatively small number of additional departments after the baseline assessment did not substantively alter the results. Another limitation is that TB transmission was not measured; therefore, whether the measures implemented affected TB transmission within the facilities could not be evaluated.

In conclusion, continuous quality improvement for TB infection prevention is imperative to manage nosocomial transmission risks. The findings from TB BASICS implementation show that it is an effective framework for promoting IPC implementation in healthcare settings. Gaps remaining after 18 months of follow-up suggest that increased investment in facilities and consideration for ongoing operation and maintenance costs may be needed to reach full implementation of all recommended IPC measures. Both funding and attention from facility leadership and local governments are needed to sustain improvements. Adoption of new national TB IPC guidelines in China may help scale the processes promoted through TB BASICS. Additional analyses are planned to report results by individual indicator to better understand the most challenging IPC indicators. This project showed that progress can be made in as few as 6 months, but facilities may benefit from developing short- and long-term improvement plans.

## Supplementary material

To view supplementary material for this article, please visit https://doi.org/10.1017/ice.2023.287
